# Clutch Frequency Affects the Offspring Size-Number Trade-Off in Lizards

**DOI:** 10.1371/journal.pone.0016585

**Published:** 2011-01-27

**Authors:** Zheng Wang, Yuan Xia, Xiang Ji

**Affiliations:** 1 Jiangsu Key Laboratory for Biodiversity and Biotechnology, College of Life Sciences, Nanjing Normal University, Nanjing, Jiangsu, People's Republic of China; 2 Hangzhou Key Laboratory for Animal Adaptation and Evolution, School of Life and Environmental Sciences, Hangzhou Normal University, Hangzhou, Zhejiang, People's Republic of China; University of Western Ontario, Canada

## Abstract

**Background:**

Studies of lizards have shown that offspring size cannot be altered by manipulating clutch size in species with a high clutch frequency. This raises a question of whether clutch frequency has a key role in influencing the offspring size-number trade-off in lizards.

**Methodology/Principal Findings:**

To test the hypothesis that females reproducing more frequently are less likely to tradeoff offspring size against offspring number, we applied the follicle ablation technique to female *Eremias argus* (Lacertidae) from Handan (HD) and Gonghe (GH), the two populations that differ in clutch frequency. Follicle ablation resulted in enlargement of egg size in GH females, but not in HD females. GH females switched from producing a larger number of smaller eggs in the first clutch to a smaller number of larger eggs in the second clutch; HD females showed a similar pattern of seasonal shifts in egg size, but kept clutch size constant between the first two clutches. Thus, the egg size-number trade-off was evident in GH females, but not in HD females.

**Conclusions/Significance:**

As HD females (mean  = 3.1 clutches per year) reproduce more frequently than do GH females (mean  = 1.6 clutches per year), our data therefore validate the hypothesis tested. Our data also provide an inference that maximization of maternal fitness could be achieved in females by diverting a large enough, rather than a higher-than-usual, fraction of the available energy to individual offspring in a given reproductive episode.

## Introduction

One of the fundamental trade-offs in life history evolution is between offspring size and number. Assuming a positive correlation between offspring size and fitness, and the finite resources available to females for reproduction, it follows that females produce either a larger number of smaller offspring or a smaller number of larger offspring. The classic offspring size theory predicts that, for a given environment, an optimal offspring size which maximizes the fitness of both mothers and offspring should be selected [1]. Following this prediction, females with different amounts of energy to invest should vary the number but not the size of their offspring and, as such, offspring size should be invariant, or vary little. However, accumulating evidence from diverse animal taxa indicates that females can adjust the size of their offspring by assessing the environment their offspring will face based on their own experience [2]−[6], or offspring size can vary in response to variation in total reproductive investment or maternal size [7]−[11].

One approach for examining how females tradeoff offspring size against number is to conduct manipulative experiments. Studies of lizards by experimental manipulation of clutch size have shown that offspring size may sometimes [12]−[15], but not always [16], vary with offspring number. For example, follicle ablation results in enlargement of offspring size in *Lacerta* (*Zootoca*) *vivipara* [12] and *Uta stansburiana* [14], but not in *Takydromus septentrionalis* [16] where females always tend to divert a fixed fraction of the available energy to individual offspring in single reproductive bouts [16]−[18]. Given that females reproduce much more frequently within a breeding season in *T. septentrionalis* (up to nine clutches) [18] than in *U. stansburiana* (up to four clutches) [19] and *L. vivipara* (single clutch for viviparous females) [20], it seems likely that clutch frequency (the number of clutches produced per year) has a key role in influencing the offspring size-number trade-off in lizards. To verify this speculation, we applied the follicle ablation technique to female Mongolian racerunners (*Eremias argus*) [15] from two populations that differ in clutch frequency to test the hypothesis that females reproducing more frequently are less likely to tradeoff offspring size against number. We formulate this hypothesis under the assumption of a positive correlation between maternal fitness and the number of surviving but not largest young [8],[21]−[23]. The largest young may not always survive best and be best able to defend resources in lizards [24], [25] and snakes [8]. Given that females reproducing more frequently are better able to increase the number of offspring produced by channeling their current surplus energy into the next clutch, we predict that they should be less likely to increase investment per offspring when undergoing follicle ablation.

## Results

Clutches reported in this paper were laid by 47 follicle-ablated (HD: 54.9±0.6 mm SVL, n = 28; GH: 59.4±0.6 mm SVL, n = 19), 40 sham-ablated (HD: 55.3±0.6 mm SVL, n = 24; GH: 58.4±0.7 mm SVL, n = 16) and 45 control (HD: 54.8±0.5 mm SVL, n = 28; GH: 59.5±0.7 mm SVL, n = 17) females. HD females were smaller than GH females (two-way ANOVA with population and surgical treatment as the factors; F_1, 126_ = 63.41, p<0.0001), but mean body sizes were similar among females in the three groups (F_2, 126_ = 0.14, p = 0.872). HD females produced their first two clutches between 29 April and 29 June, and GH females between 20 May and 5 July. Mean clutch intervals differed between the two populations (two-way ANOVA with population and surgical treatment as the factors; F_1, 126_ = 24.16, p<0.0001), but not among the three groups (F_2, 126_ = 0.49, p = 0.614). HD females produced a second clutch on average 19.9±0.5 days after the first clutch, and GH females, on average 24.5±0.8 days.

**Figure 1:**
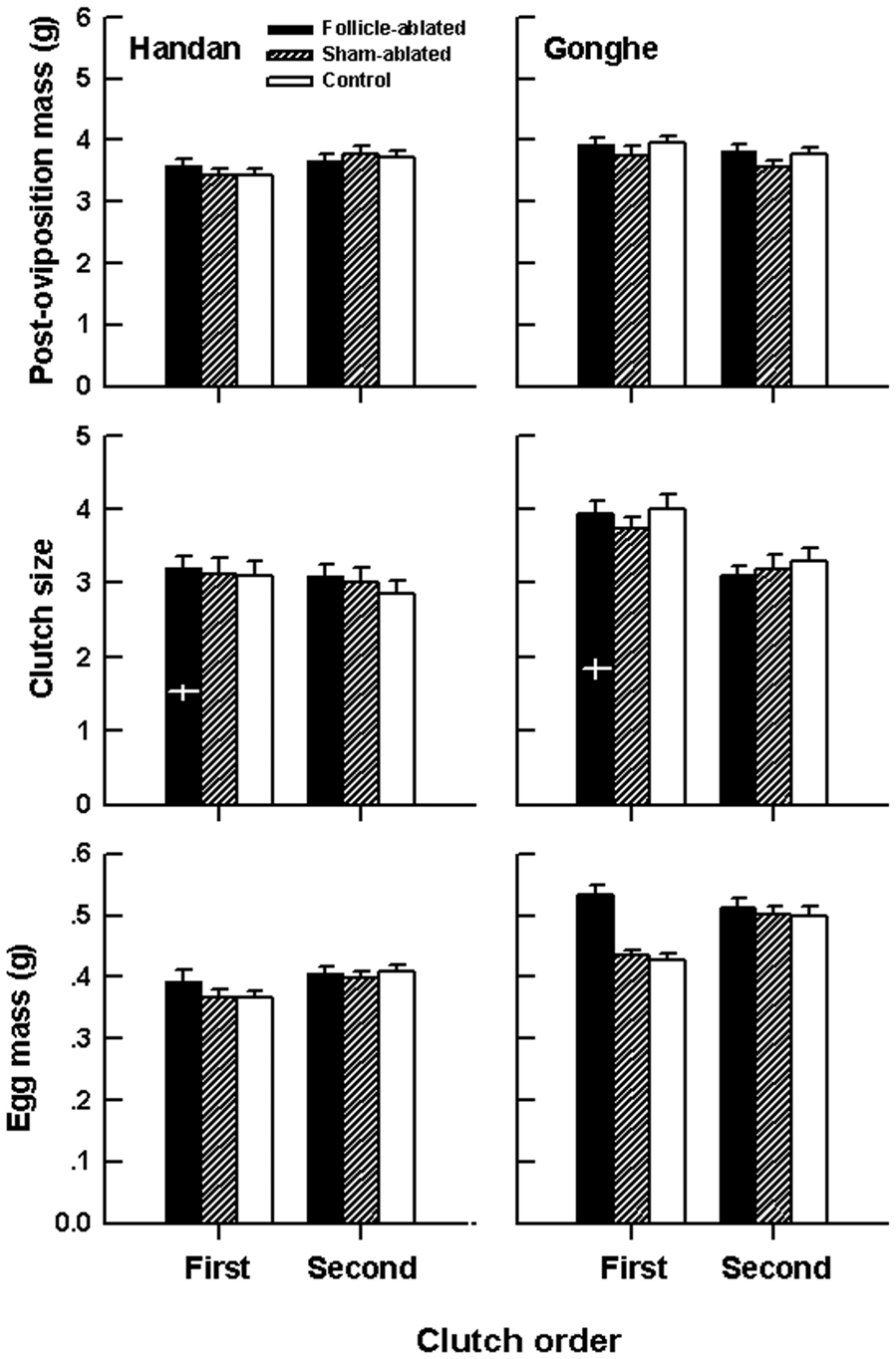
Mean values (+SE) for post-oviposition body mass, clutch size and egg mass of the females involved. Clutch size for the follicle-ablated females was calculated as the sum of yolking follicles removed and eggs produced. Black, diagonal and open bars represent follicle-ablated, sham-ablated and control females, respectively. White horizontal and vertical lines represent mean and ±SE for the actual number of eggs laid by follicle-ablated HD and GH females, respectively.

Figure 1 shows mean values (+SE) for post-oviposition body mass, clutch size and egg mass of the experimental females. Of the three variables, only egg size was independent of maternal SVL within each population × treatment × clutch combination (linear regression analysis; all r^2^ <0.091, and all p>0.217). Sham-ablated females did not differ from control females of the same population in any of the traits examined (Table 1). Data of these two groups showed that HD females laid smaller eggs than did GH females (one-way ANOVA; 1^st^ clutch-F_1, 83_ = 32.39, 2^nd^ clutch-F_1, 83_ = 63.63, both p<0.0001), but they did not differ from GH females of the same SVL in clutch size (one-way ANCOVA with SVL as the covariate; 1^st^ clutch-F_1, 82_ = 0.02, 2^nd^ clutch-F_1, 82_ = 0.25, both p>0.620), in each of the two successive clutches. Follicle-ablated HD females produced an average of 1.5±0.1 eggs in the first clutch (the first post-surgical clutch), and follicle-ablated GH females produced an average of 1.8±0.1 eggs; their original clutch sizes, after adding the ablated follicles, did not differ from those in the other two groups (Table 1). Follicle ablation resulted in a substantial increase in egg size in GH females, but not in HD females (Table 1). HD females gained mass during the time interval between the first and second clutches, whereas GH females lost mass (Table 1). GH females switched from producing a larger number of smaller eggs in the first clutch to a smaller number of larger eggs in the second clutch. HD females also laid smaller eggs in the first clutch and larger eggs in the second clutch, but they kept clutch size constant between the two successive clutches (Table 1).

**Table 1:** Results of repeated-measures ANOVA with clutch order as the within subject factor and surgical treatment as the between subject factor.

	Clutch order	Surgical treatment	Interaction
Handan population			
Post-oviposition mass	F_1, 77_ = 26.46, p<0.00011^st^ <2^nd^	F_2, 77_ = 0.05, p = 0.950	F_2, 77_ = 3.26, p = 0.044
Clutch size	F_1, 77_ = 1.54, p = 0.218	F_2, 77_ = 0.44, p = 0.647	F_2, 77_ = 0.13, p = 0.882
Egg mass	F_1, 77_ = 12.02, p<0.0011^st^ <2^nd^	F_2, 77_ = 0.94, p = 0.396	F_2, 77_ = 1.12, p = 0.330
Gonghe population			
Post-oviposition mass	F_1, 49_ = 10.02, p<0.0031^st^ >2^nd^	F_2, 49_ = 1.48, p = 0.237	F_2, 49_ = 0.24, p = 0.791
Clutch size	F_1, 49_ = 28.52, p<0.00011^st^ >2^nd^	F_2, 49_ = 0.49, p = 0.616	F_2, 49_ = 0.38, p = 0.687
Egg mass	F_1, 49_ = 12.23, p<0.0011^st^ <2^nd^	F_2, 49_ = 11.44, p<0.0001F > (S = C)	F_2, 49_ = 7.28, p<0.002

F: follicle-ablated females; S: sham-ablated females; and C: control females. Tukey's *post hoc* comparison was performed on the trait that differed among females of the three treatments.

## Discussion

Under the conditions described above, HD females produce an average of 3.1 clutches per year between late April and mid-July, and GH females produce an average of 1.6 clutches per year between mid-May and early July [26]. Given that the unlimited food availability, the absence of predators, and the suitable thermal environments often result in lizards under laboratory conditions to produce as many clutches as they can [18], it seems likely that the differences in the length of breeding season and clutch frequency between HD and GH females are determined ultimately by natural selection, presumably as a consequence of their adaptation to local environmental conditions (e.g., lower monthly mean temperatures, lower monthly rainfall, and shorter periods of time suitable for foraging in GH [26]) at the population level. The time interval between the first and second clutches was longer in GH females that produced larger eggs but did not differ from HD females of the same SVL in clutch size, suggesting that female *E. argus* took a longer time to produce larger eggs. The longer breeding season and the shorter clutch interval explain why HD females reproduce more frequently than do GH females.

Our manipulation had the desired effect of reducing clutch size in the first but not the second post-surgical clutch. Follicle-ablated females did not change clutch size upward or downward, and even after adding the removed follicles, they did not differ from sham-manipulated and control females in clutch size. Follicle-ablated females produced a second clutch as normally as did females in the controls, indicating that follicle ablation did not affect clutches subsequent to the post-surgical clutch. Consistent with the study of *T. septentrionalis* [16], HD females did not change the size of their eggs following follicle ablation; consistent with the results reported for *L. vivipara* [12] and *U. stansburiana* [14], GH females produced larger eggs (and thus, larger hatchlings [27]). What can be concluded from these observations is that females reproducing more frequently are less likely to tradeoff offspring size against number.

The relationship between offspring size and number is the net outcome of complex interactions between numerous factors that affect offspring size and fecundity [28], [29]. Once clutch size is fixed, the energy available for vitellogenesis until the time of ovulation determines final egg size in lizards [30]. In our study, egg size was unlikely to be constrained by energy availability because females produced clutches under the laboratory conditions without any food limitation. Moreover, total reproductive allocation of resources was unlikely to be constrained by the amount of space available to hold eggs within a follicle-ablated female's abdomen because of the reduced clutch mass and therefore clutch volume. So, why did follicle ablation result in enlargement of egg size in GH females but not in HD females? Our explanation is that females tend to divert their current surplus energy not used in the current reproduction to production of larger eggs in the GH population, but to production of an additional clutch in the HD population. GH females can produce a maximum of two clutches per year, whereas ∼74% HD females are able to produce at least two clutches per year [26]. Thus, compared with HD females, GH females have a more limited opportunity to enhance their fitness by increasing the number of clutches and therefore the total number of eggs produced per year. However, by diverting a larger fraction of their current available energy to individual eggs, GH females can enhance the fitness of their offspring. Increasing the amount of resources invested per offspring when possible is ecologically important for females in the colder, GH population where the season suitable for growth is shorter. The lack of response to follicle ablation in HD females cannot be due to their smaller size and inability to produce larger eggs, because egg size is independent of maternal size (SVL) within each clutch × treatment combination (linear regression analysis; all r^2^ <0.049, and all p>0.256). HD females have a more ample opportunity to produce an additional clutch and therefore are better able to increase the number of eggs produced per year. Thus, diverting a large enough, rather than a higher-than-usual, fraction of the available energy to individual eggs could be a reproductive strategy adopted by HD females to maximize their own fitness. In the HD population, most probably, selection has maximized maternal fitness to achieve an optimal balance between egg size and number in single reproductive bouts.

GH females switched from producing more smaller eggs early in the breeding season to fewer larger eggs later in the season, showing that they cannot increase the size of their eggs without a concomitant reduction in the number of eggs produced. Dividing accessible resources into smaller amounts for each egg allows females to increase the number of eggs produced. Hatchlings from eggs produced early in the breeding season have a longer growth period than progenies from later clutches. Thus, producing a larger number of smaller eggs early in the breeding season could be a reproductive tactic adopted by females to enhance their fitness. HD females showed a similar pattern of seasonal shifts in egg size, but they kept clutch size constant between the first two successive clutches. This suggests that egg size is insensitive to variation in clutch size in the HD population and, more interestingly, adds evidence that females reproducing more frequently are less likely to tradeoff offspring size against number.

## Materials and Methods

*Eremias argus* is an oviparous lacertid lizard with a distribution covering North China (southwards to Jiangsu and westwards to Qinghai), Russia (region of Lake Baikal), Mongolia and Korea [31]. We collected pre-productive females and adult males between 10 April and 6 May 2008 from two populations, one in Handan (HD: 36°36′N, 114°28′E, ∼70 m elevation) and the other in Gonghe (GH: 36°03′N, 101°13′E, ∼2300 m elevation). The annual mean temperature is about 7°C higher in the HD population (∼13°C) than in the GH population (∼6°C); depending on SVL (snout-vent length), HD females produce 1−5 clutches per breeding year between late April and mid-July, and GH females produce 1−2 clutches between mid-May and early July [26]. Embryonic stages at oviposition fall within the range of Stage 24−27 in the Dufaure and Hubert's (1961) developmental series [32], with a mode of Stage 26 in both populations [26]. Ten to 12 lizards, 5−6 of each sex, were housed in each 900×650×600 mm (length × width × height) communal cage. Mealworm larvae (*Tenebrio molitor*) and house crickets (*Achetus domestica*) dusted with multivitamins and minerals were provided daily, so that excess food was always available in the cages. Fresh water was also provided daily. Thermoregulatory opportunities were provided during daytime hours by a 100 W incandescent lamp; overnight temperatures followed indoor temperatures (22−28°C).

We randomly allocated females at early stages of vitellogenesis to follicle-ablated and sham-ablated groups, and kept the remaining females as controls. We anaesthetized females by moving them into a 500 ml jar with an ether-saturated tampon. The anesthesia was often obtained in less than 9 min and lasted 20–30 min; the time of recovery from anesthesia ranged from 30–45 min. With this technique no deaths were observed. We taped the anaesthetized female to a sterile board, and then made a 5−7 mm incision, 2−3 mm to the left of the mid-ventral line with a sterile scalpel. We lifted the left-sided ovary of the follicle-ablated female out of the incision with a pair of forceps, counted and measured yolking follicles, and then ablated them with sterile syringes. All yolking follicles in the left-sided ovary, measuring 2.5−4.0 mm diameter, were ablated with the follicle theca left intact. The number of ablated follicles, ranging from one to three, corresponded roughly to 50% of the expected clutch (Fig. 1). The incision was closed using a surgical suture and cleaned daily with 75% alcohol. After a 5-day recovery, females were moved back into the communal cages. The sham-ablated females underwent the same protocol without any follicle ablation. Our experimental procedures complied with the current laws on animal welfare and research in China, and were approved by the Animal Research Ethics Committee of Nanjing Normal University (Permit No. AREC 2008-04-016).

Females with shelled oviductal eggs were housed individually in 200×150×200 mm egg-laying cages with 40 mm depth moist soil and a 20 W spotlight mounted in each cage to allow thermoregulation. Eggs were collected and weighed less than 3 h post-laying, to minimize water uptake or loss between the egg and the substrate [26]. Post-oviposition females were measured and weighed before they were returned to the communal cages where they remained until they again carried shelled oviductal eggs, at which time they were once again transferred to the egg-laying cages.

We ended our experiment after the first two clutches were collected, and excluded females laying a single clutch or abnormal eggs with condensed yolk from analyses. Because of maternal effects, it is not valid to treat eggs within the same clutch as independent for the purposes of statistical analysis; thus, our statistical analyses are based upon mean values for egg sizes per clutch. We used linear regression analysis, one-way ANOVA, two-way ANOVA, one-way ANCOVA (to correct for maternal SVL and to test for homogeneity of slopes), repeated-measures ANOVA and Tukey's *post hoc* test to analyze the corresponding data. No data required transformation to meet the assumptions for parametric tests. All statistical analyses were performed with the Statistica software (version 6.0 for PC, Tulsa, OK, USA). Throughout this paper, values are presented as mean ± SE, and the significance level is set at α = 0.05.
